# Birth and Early Childhood Outcomes in Families Receiving an Unconditional Prenatal Cash Benefit

**DOI:** 10.1001/jamanetworkopen.2025.26996

**Published:** 2025-08-14

**Authors:** Jennifer E. Enns, Marni Brownell, Nathan C. Nickel, Mariette Chartier, Dan Chateau, Joykrishna Sarkar, Hera J. M. Casidsid, Farzana Quddus, Elaine Burland, Alan Katz, Robert G. Santos, Wanda Phillips-Beck, Julianne Sanguins, Hannah Owczar

**Affiliations:** 1 Manitoba Centre for Health Policy, Department of Community Health Sciences, Rady Faculty of Health Sciences, University of Manitoba, Winnipeg, Manitoba, Canada; 2 Ageing and Aged Care Analysis Unit, Community Services Group, Australian Institute of Health and Welfare, Canberra, Australian Capital Territory, Australia; 3 Early Childhood Development Research, Research Partnerships and Innovation, Red River College Polytechnic, Winnipeg, Manitoba, Canada; 4 First Nations Health and Social Secretariat of Manitoba, Winnipeg, Manitoba, Canada; 5 University of Manitoba, Winnipeg, Manitoba, Canada

## Abstract

**Importance:**

The Canadian province of Manitoba has offered an unconditional cash benefit (maximum CAD $81.41/mo) to low-income pregnant women since 2001. Evaluations have found the benefit was associated with improved birth and child development outcomes, but its effectiveness may be diminishing because the benefit amount has not changed in 20 years.

**Objective:**

To examine whether the Healthy Baby Prenatal Benefit remains an effective strategy for supporting maternal and child health.

**Design, Setting, and Participants:**

This retrospective cohort study was conducted among mothers with a singleton birth in Manitoba, Canada, from 2003 to 2019. Low-income mother-child dyads were identified using income assistance records. Data analyses were conducted from June to December 2024.

**Exposure:**

Receipt of the benefit during the second and third trimesters of pregnancy.

**Main Outcomes and Measures:**

Propensity scores and inverse probability of treatment weights were developed from sociodemographic information, which were used to reduce standardized differences between benefit recipients and nonrecipients. Risk ratios (RRs) for birth outcomes and developmental vulnerability in 5 domains at kindergarten were calculated.

**Results:**

The 17 970 benefit recipients (mean [SD] age at first birth, 19.52 [3.53] years) and 8301 nonrecipients (mean [SD] age at first birth, 19.38 [3.43] years). After applying inverse probability of treatment weights, all standardized differences were less than 0.10. From 2003 to 2019, the RRs increased among recipients vs nonrecipients for low birth weight (2003: RR, 0.68 [95% CI, 0.56-0.82]; 2019: RR, 0.92 [95% CI, 0.76-1.11]), preterm birth (2003: RR, 0.72 [95% CI, 0.62-0.83]; 2019: RR, 0.95 [95% CI, 0.83-1.09]), and small-for–gestational age (2003: RR, 0.83 [95% CI, 0.71-0.96]; 2019: RR, 0.97 [95% CI, 0.83-1.43]) and decreased for large-for–gestational age (2003: RR, 1.13 [95% CI, 1.01-1.26]; 2019: RR, 1.11 [95% CI, 0.99-1.24]) and breastfeeding initiation (2003: RR, 1.06 [95% CI, 1.02-1.10]; 2019: RR, 1.01 [95% CI, 0.98-1.05]). The RR for developmental vulnerability in kindergarten increased in the domains of language and cognitive development (2003: RR, 0.77 [95% CI, 0.66-0.89]; 2019: RR, 1.07 [95% CI, 0.94-1.21]) and communication and general knowledge (2003: RR, 0.83 [95% CI, 0.72-0.96]; 2019: RR, 0.93 [95% CI, 0.82-1.05]).

**Conclusions and Relevance:**

In this cohort study of low-income mothers and newborns, the associations of the Healthy Baby Prenatal Benefit with birth and child development outcomes diminished from 2003 to 2019, with many disappearing. Given the importance of prenatal health to these outcomes, these findings suggest that the benefit amount should be increased and adjusted for inflation in future years.

## Key Points

**Question:**

Do participants in the Healthy Baby Prenatal Benefit Program still have improved birth and early childhood outcomes compared with nonparticipants, despite no change in the benefit amount over the last 20 years?

**Findings:**

In this cohort study with 17 970 low-income mothers and children, receiving the benefit was initially associated with better birth outcomes. However, many of these associations weakened over time and were no longer statistically significant or practically meaningful.

**Meaning:**

The findings of this study suggest that government funders should regularly adjust child benefit amounts for inflation to maintain improvements to birth and early childhood outcomes for recipients.

## Teaser

This cohort study evaluates whether the Health Baby Prenatal Benefit, an unconditional cash benefit given to low-income pregnant women in Manitoba, Canada, is associated with improved birth and childhood outcomes, despite the benefit amount not changing in 20 years.

## Introduction

The prenatal period is a critical time for fetal and newborn health and ongoing early childhood development.^1^ Inadequate prenatal care and nutrition, poor maternal health, and maternal exposure to high levels of stress and harmful substances can put the developing fetus at risk for adverse outcomes, such as preterm birth or low birth weight.^1,2,3^ Brain development in utero and continuing after birth may also be compromised,^4,5^ affecting children’s cognitive and executive functioning and potentially leading to longer-term physical or behavioral issues.^6^ Children born into socioeconomically disadvantaged households are at higher risk for these adverse outcomes throughout childhood.^4,7^ A 2020 systematic review^8^ presented strong evidence that increasing household income for families living in poverty improved children’s health and cognitive and social-behavioral development and also had a positive effect on intermediate outcomes, such as good maternal mental health, positive parenting, and a stable home environment. Early intervention for low-income families is therefore a compelling strategy to promote maternal and infant health and healthy child development.

International recognition of the link between poverty and poor infant and child health has led many countries to establish programs to aid low-income families expecting or raising children. These programs differ in the supports they offer, such as health care, nutritional supplements, social supports, or some type of cash benefit. Their specific impacts on outcomes vary, but there is good evidence that such supports do indeed make a difference for recipient families, and for children in particular. A 2024 review^9^ provides an overview of the government-funded child cash benefit programs operating in 140 countries across the globe. Two well-known and long-standing programs that have been shown to have impacts on child health are *Oportunidades *in Mexico, also known as* Progresa *(1997-2002) and *Prospera *(2019-2019), and *Bolsa Familia* in Brazil, both of which provide conditional cash transfers to participating low-income families for meeting specified health care visit and school attendance requirements.^10,11^ These programs have resulted in higher birth weights and lower childhood illness rates^12^; improved physical size (height-for-age, less stunting and overweight)^10^; better outcomes in motor, cognitive, and language development for children ranging from 2 to 4 years of age; and lower mortality.^11,13^ The *Oportunidades *program is no longer operating as of 2019. An unconditional cash transfer program for low-income families in the United States, Baby’s First Years,^14^ has so far had mostly non–statistically significant results for the outcomes measured (maternal assessments of children’s overall health, sleep, and health care utilization^15^ and maternal use of alcohol, cigarettes, or opioids or household expenditures on alcohol or cigarettes^16^), despite the cash transfer being quite substantial. One study^17^ did show a change in infant brain activity associated with the development of subsequent cognitive skills, but the otherwise largely disappointing results from this program could be in part because the cash transfer is only offered postnatally. This would suggest that not only the dosage but also the timing of the benefit (prenatal or postnatal) are important factors. In their review, Shaefer et al^9^ also highlight unconditional prenatal cash transfer programs in high-income countries, noting that these transfers resulted in lower rates of low birth weight, among other outcomes, in populations in the United States,^18,19,20,21^ England and Wales,^22^ South Korea,^23^ and Spain.^24^

In Canada, financial supports for families exist in the form of federal child benefits, which are also only available after the birth of the child. However, the province of Manitoba is a long-standing exception, having offered prenatal financial supports to low-income families through the provincial Healthy Baby Program since 2001.^25^ The program provides an unconditional income supplement, called the Healthy Baby Prenatal Benefit (maximum CAD $81.41/mo), to low-income pregnant women during their second and third trimesters of pregnancy, along with informational brochures about prenatal nutrition, breastfeeding, and healthy infant development. Evaluations of the program have found that it was associated with reduced rates of preterm birth and low birth weight^26,27,28^ and with increased breastfeeding initiation among mother-newborn pairs.^26,28^ In a First Nations population, recipients’ offspring also had better child development scores in kindergarten compared with those who were not enrolled in the program.^28^ Despite these successes, however, there is concern among program administrators that the positive outcomes associated with this program may be diminishing because the amount of the benefit has not changed in the last 20 years and therefore has not kept pace with the rising cost of living. This concern was also highlighted by program recipients in a qualitative study.^29^ Therefore, we conducted an analysis of recipients’ birth outcomes and early childhood development over time (2003-2019) to evaluate whether the Healthy Baby Prenatal Benefit remains associated with maternal and infant health and child development.

## Methods

### Study Setting

The study was conducted in Manitoba, Canada (population 1.4 million). The major urban center is Winnipeg (population 800 000), and the remaining residents live in smaller cities and towns or in rural and remote communities. In the 2021 Canada Census, 5.0% of the national population identified as Indigenous; in Manitoba that year, Indigenous peoples made up 18.1% of the population, the highest proportion among provinces and territories after the Northwest Territories, Yukon, and Nunavut.^30^ Manitoba is otherwise broadly representative of other provinces on key health and social indicators.^31,32^

### Ethics and Reporting

This study received approval from the University of Manitoba’s Human Research Ethics Board and the Manitoba government’s Health Information Privacy Committee. With regard to privacy and confidentiality, under provincial legislation, individual patients or participants must give consent for disclosure and use of their data in research when direct contact with these individuals is anticipated. However, the University of Manitoba Human Research Ethics Board waived the requirement for individual consent because the study constitutes a secondary use of data, and therefore, there is no direct contact with patients or participants. Additionally, several measures (such as deidentification of the data and both physical and digital limitations to accessing the data) have been taken to protect individual privacy. The reporting of the study adheres to the Strengthening the Reporting of Observational Studies in Epidemiology (STROBE) reporting guidelines for cohort studies.^33^

Analyses for this study were initially conducted in 2019 to 2020, but COVID-19 pandemic–related delays prevented us from finalizing the article at that time. We resumed work on the analyses from June to December 2024, at which time we updated the cohort and outcomes with the most recent years of data available.

### Intervention

The Healthy Baby Program was established by the Manitoba government in 2001 with the aim of improving prenatal health and birth outcomes.^34^ To enroll for the prenatal benefit, women must complete an application providing proof of pregnancy and have an annual income of CAD $32 000 or less. Health care practitioners and the provincial government department responsible for the program share information about it continuously in health care appointments and in public awareness campaigns. The application form has been designed to be as simple as possible, and program staff can help applicants fill it out over the phone. In Manitoba’s universal health system, all residents are able to see a primary care physician or nurse practitioner for confirmation of pregnancy without referral or out-of-pocket payment.

Upon enrollment, program recipients receive monthly mailings starting in their second trimester. They receive the Healthy Baby Prenatal Benefit in the form of a check (on a sliding scale based on their income to a maximum of CAD $81.41/mo) as well as pamphlets about prenatal nutrition, breastfeeding, and healthy infant development. There are no conditions placed on how recipients spend the money.

### Data Sources

Data were obtained from the Manitoba Population Research Data Repository at the Manitoba Centre for Health Policy, University of Manitoba. The repository contains whole-population health and social data for more than 99% of the entire province, except for federally insured individuals (eg, military personnel, individuals incarcerated in federal prisons). All personal information in these files (eg, names and addresses) is removed, but the records can be linked together at the individual level using a scrambled numeric identifier. In this way, records can be linked across different services, across sectors, and over time. The repository data have been used extensively in population and public health research studies, and their quality and validity have been well documented.^35,36,37,38,39,40^ We also used administrative data from the Healthy Baby Program (Prenatal Benefit and Community Support Program), the Families First Screen (a newborn risk screen administered by public health nurses after each birth in Manitoba), the province’s population health insurance registry, hospital discharge abstracts, physician visit records, income assistance data, and scores from the Early Development Instrument (EDI), which assesses population-level vulnerabilities in child development in kindergarten.^41^

### Study Population

**Figure 1.**
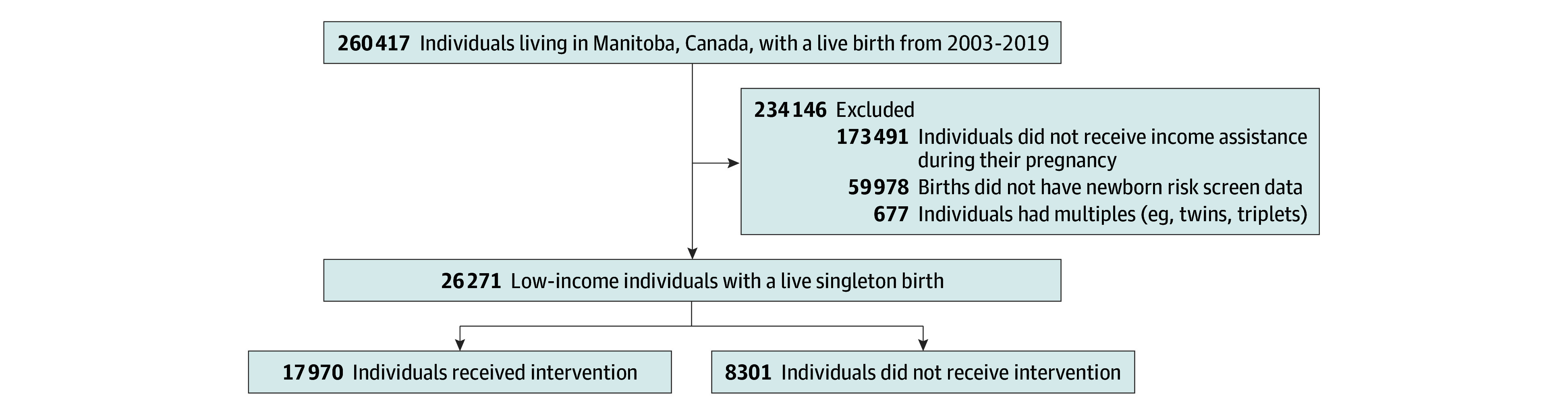
Study Flowchart

The study population included all low-income women in Manitoba who had a live singleton birth from January 1, 2003, to July 31, 2019 (Figure 1). We excluded women for whom we did not have newborn risk screen data and women who had multiples (eg, twins, triplets). To ensure we were including only low-income women in the study, we limited the population to women who received income assistance during pregnancy. We used this approach because we did not have access to whole-population family income data needed to calculate the national measures, such as the Low Income Measure or Low Income Cut-Off. We then divided the women into 2 study groups: low-income women who had received the Healthy Baby Prenatal Benefit (n = 17 970) and those who did not receive the benefit (n = 8301).

### Main Outcomes and Measures

We examined birth outcomes (low birth weight, preterm birth, small- and large-for–gestational age births, and breastfeeding initiation) using data from hospital discharge abstracts. We examined childhood development at kindergarten using data from the EDI. Definitions of the outcomes are provided in Table 1.^41,42^ Since preliminary analyses showed that nearly all program recipients in our study (99.1%) received the maximum benefit amount, we did not test for a dose-response association.

**Table 1.** Study Outcome Definitions

Outcome	Definition
Birth outcomes	
Low birth weight	<2500 g
Preterm birth	<37 wk Gestational age
Small for gestational age	<10th Percentile for gestational age and sex, based on Kramer et al,^42^ 2001
Large for gestational age	>90th Percentile for gestational age and sex, based on Kramer et al,^42^ 2001
Breastfeeding initiation	Exclusive or partial breastfeeding at hospital discharge
Early childhood development outcomes^a^	
Physical health and well-being	Gross and fine motor skills, physical independence, and readiness for the school day
Social competence	Overall social competencies, capacity for respect and responsibility, approaches to learning, and readiness to explore new things
Emotional maturity	Prosocial and helping behaviors as well as hyperactivity and inattention, aggressive, anxious and fearful behaviors
Language and cognitive development	Basic and advanced literacy skills, numeracy skills, interest in math and reading, and memory
Communication skills and general knowledge	English language skills and general knowledge

^a^ Measured using the Early Development Instrument, which measures the rate of the developmental vulnerability of kindergarten children at the population level.^41^

### Statistical Analysis

To determine whether the groups were comparable at baseline, we examined their characteristics using data from the newborn risk screen and several additional variables from other repository data files. In assessing differences between groups, *P* values were considered statistically significant at *P* < .001. Because we observed significant differences for many of the characteristics, we adjusted for differences using multivariable logistic regression to develop propensity scores using receipt of the benefit as the dependent variable and the aforementioned characteristics as covariates.^43^ Each woman’s propensity score indicates the probability that she received the benefit given her observed characteristics. From the propensity scores, we then developed inverse probability of treatment weights (IPTWs), which we used to reduce differences between groups.^44,45^ Here we report the mean crude and weighted standardized differences for the study period as a whole; we also examined the standardized differences in 4-year periods to assess how the benefit recipients’ characteristics may have changed over the last 2 decades (eFigure in Supplement 1).

Generalized linear models with a binomial distribution and a log link function were used to generate risk ratios (RRs) with 95% CIs. We calculated risk ratios for the entire study period of 2003 to 2019 and for each year between study groups. The results were adjusted for birth year, which was included in the models as a linear and quadratic continuous variable and as an interaction with receipt of the benefit. Data analysis was conducted in SAS version 9.4 (SAS Institute).

## Results

**Figure 2.**
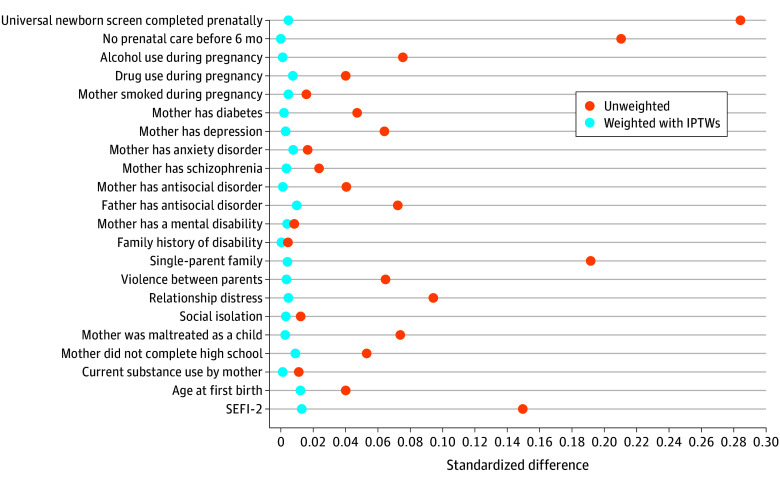
Standardized Differences in Study Population Characteristics Before and After Applying Inverse Probability Treatment Weights (IPTWs), 2003-2019 SEFI-2 indicates Socioeconomic Factor Index.

The cohort comprised 17 970 low-income mother-child dyads (mean [SD] mothers’ age at first birth, 19.52 [3.53] years) who received the benefit and 8301 mother-child dyads (mean [SD] mothers’ age at first birth, 19.38 [3.43] years) who were eligible for the benefit but did not apply and therefore did not receive the benefit. Study population characteristics are presented in Table 2. At baseline, there were some differences between groups, but overall both experienced a similar degree of challenges. For example, a higher proportion of women who received the benefit consumed alcohol during their pregnancy, had been diagnosed with depression, and were single parents. On the other hand, a higher proportion of women who did not receive the benefit were younger at their first birth and did not receive any prenatal care before their third trimester. To account for these differences, we applied IPTWs to balance the groups before examining outcomes. As shown in Figure 2, before we applied the IPTWs, the standardized differences between groups were as high as 0.29; afterwards, all covariates had standardized differences that were less than 0.10.

**Table 2.** Sociodemographic Characteristics of the Study Population

Characteristic	Women, No. (%)		*P* value
	Received HBPB (n = 17 970)	Did not receive HBPB (n = 8301)	
Universal newborn screen completed prenatally^a^	3017 (16.8)	630 (7.6)	<.001
No prenatal care before 6 mo	949 (5.3)	914 (11.0)	<.001
Alcohol use during pregnancy	4218 (23.5)	1689 (20.4)	<.001
Drug use during pregnancy	2917 (16.2)	1226 (14.8)	.002
Mother smoked during pregnancy	8753 (48.7)	4109 (49.5)	.24
Mother has diabetes	343 (1.9)	109 (1.3)	<.001
Mother has depression	4561 (25.4)	1879 (22.6)	<.001
Mother has anxiety disorder	2194 (12.2)	969 (11.7)	.21
Mother has schizophrenia	313 (1.7)	120 (1.5)	.07
Mother has antisocial disorder	588 (3.3)	214 (2.6)	.002
Father has antisocial disorder	1040 (5.8)	349 (4.2)	<.001
Mother has a mental disability	297 (1.7)	128 (1.5)	.52
Family history of disability	755 (4.2)	355 (4.3)	.76
Single parent family	8584 (47.8)	3181 (38.3)	<.001
Violence between parents	1527 (8.5)	564 (6.8)	<.001
Relationship distress	3147 (17.5)	1167 (14.1)	<.001
Social isolation	1405 (7.8)	622 (7.5)	.36
Mother was maltreated as a child	3675 (20.5)	1457 (17.6)	<.001
Mother did not complete high school	9258 (51.5)	4056 (48.9)	<.001
Current substance use by mother	633 (3.5)	275 (3.3)	.40
Age at first birth, mean (SD), y^b^	19.52 (3.53)	19.38 (3.43)	.003
Area-level SES index, mean (SD)^c^	0.91 (0.97)	0.76 (0.98)	<.001

Abbreviations: HBPB, Healthy Baby Prenatal Benefit; SES, socioeconomic status.

^a^ The Families First universal newborn screen is typically completed postnatally, so when a screen is completed prenatally, this indicates the presence of additional risk factors.

^b^ Data were available for 17 956 women who received HBPB and 8299 who did not.

^c^ Data were available for 17 947 women who received HBPB and 8283 who did not.

To determine whether there were changes over time to the women’s characteristics that might differentially affect the 2 groups, we also present these standardized differences in 4-year time periods (eFigure in Supplement 1). Although each period saw only minor changes in differences between groups, it appears that there were more differences prior to weighting in the most recent 4-year period (2014-2019), and prenatal screening remained different between the groups after weighting. This could be the result of changes in practice or in program implementation, or it could reflect a growing gap in health equity between the 2 study groups.

**Figure 3.**
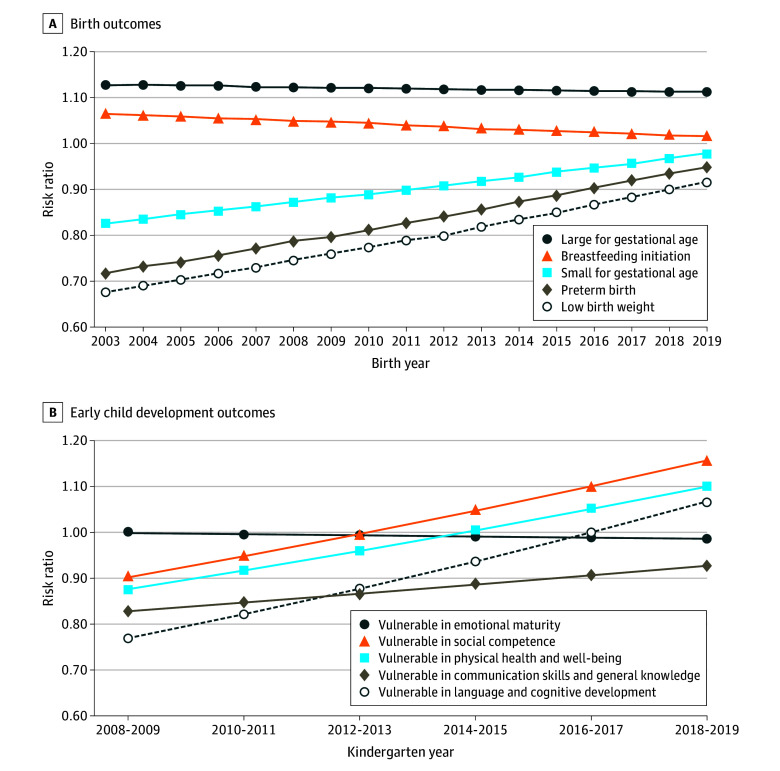
Association of Receiving the Healthy Baby Prenatal Benefit With Birth and Early Childhood Development Outcomes

Figure 3A shows risk ratios for the birth outcomes of women who did and did not receive the benefit. At the start of the study period (2003), receiving the benefit was associated with a higher likelihood of breastfeeding initiation (RR, 1.06 [95% CI, 1.02-1.10]) and large-for–gestational age births (RR, 1.13 [95% CI, 1.01-1.26]) and a lower likelihood of preterm (RR, 0.72 [95% CI, 0.62-0.83]), low birth weight (RR, 0.68 [95% CI, 0.56-0.82]), and small-for–gestational age births (RR, 0.83 [95% CI, 0.71-0.96]). Over time, the strength of these associations weakened until they were no longer statistically significant. By the end of the study period (2019), none of the birth outcomes were associated with receipt of the benefit (breastfeeding initiation: RR, 1.01 [95% CI, 0.98-1.05]; large-for–gestational age birth: RR, 1.11 [95% CI, 0.99-1.24]; preterm birth: RR, 0.95 [95% CI, 0.83-1.09]; low birth weight: 0.92 [95% CI, 0.76-1.11]; small-for–gestational age birth: RR, 0.97 [95% CI, 0.83-1.43]).

Figure 3B shows risk ratios for EDI scores for the 5 developmental domains for the children of women who did and did not receive the benefit. At the start of the study period (birth year 2003, EDI data collection year 2008-2009), receiving the benefit was associated with lower developmental vulnerability for kindergarten children in the domains of language and cognitive development (RR, 0.77 [95% CI, 0.66-0.89]) and communication skills and general knowledge (RR, 0.83 [95% CI, 0.72-0.96]). However, by birth year 2013 (EDI data collection year 2018-2019), this association was no longer significant (language and cognitive development: RR, 1.07 [95% CI, 0.94-1.21; communication and general knowledge: RR, 0.93 [95% CI, 0.82-1.05]). Receipt of the benefit was not associated with any of the other 3 domains in any of the EDI collection years.

All numerical values for Figures 3A and B are available in eTables 1 and 2 in Supplement 1. Risk differences are presented in eTables 3 and 4 in Supplement 1.

## Discussion

This retrospective cohort study examined whether newborn and early childhood development outcomes associated with receiving the Healthy Baby Prenatal Benefit changed from 2003 to 2019. The study showed that receiving the benefit was initially associated with better health outcomes for newborns and mothers, including a lower likelihood of preterm birth, low birth weight, and small-for–gestational age births and a higher likelihood of breastfeeding initiation. Children whose mothers received the benefit were also initially less likely to be vulnerable in 2 domains of development in kindergarten: language and cognitive development as well as general knowledge and communication skills. However, many of the associations have weakened over time, and the cash benefit, the dollar amount of which has stayed the same over nearly 2 decades, was no longer associated with these outcomes in the last several years of the study period.

Our findings align with many other research studies demonstrating that unconditional cash transfer programs are beneficial to recipients and can address poverty-related harms to maternal and child health.^17,26,27,28,46,47,48^ Of particular note, 2 global systematic reviews^47,49^ that examined unconditional cash transfers concluded that they yielded the greatest impacts on birth weight and infant mortality outcomes. However, our results suggest that the Healthy Baby Prenatal Benefit is no longer keeping pace with inflation and other economic forces and therefore has not maintained its impact over time. The maximum monthly benefit amount of CAD $81.41 in 2001 would be equivalent to CAD $112.94 in 2019 (accounting for inflation^50^), which suggests an average annual increase of 1.84% from 2001 on would have been needed to keep up with inflation to 2019. Many other programs face challenges associated with declining purchasing power: Shahidi et al^51^ studied social welfare policy and programs in high-income countries and reported that the scope and generosity of many current programs are inadequate to offset existing socioeconomic disadvantages associated with health outcomes.

The initial evaluations of the Healthy Baby Prenatal Benefit and the finding that a prenatal cash benefit of only CAD $81.41/mo was associated with improved birth outcomes^25,26,27,28^ was of great interest to researchers and policymakers, given that measurably changing clinical outcomes for people living in poverty is often difficult. Many policy interventions focus attention on the individual behaviors of pregnant women (such as adherence to diet or exercise patterns),^52^ but in a context that recognizes the close relationship between low income and health disparities, the fundamental causes of poor health outcomes must be addressed if there is to be any hope of improving outcomes at the population level.^53^ The mechanisms by which the benefit works are reported by Struthers et al,^29^ who interviewed recipients of the benefit about how they used the money. Participants in that study described how the money helped them prepare for the birth of their child, improve their nutrition, and engage in self-care behaviors to reduce stress.^29^ The findings align with the developmental origins of health and disease hypothesis, ie, that environmental influences in prenatal and early infant phases of life are linked to the risk of developing metabolic disorders and other chronic illnesses later in life.^1,54^ Through epigenetic pathways, low socioeconomic status is thought to increase exposure to stress and influence pregnancy and birth outcomes, elevating the risk of preeclampsia, eclampsia, gestational diabetes, low birth weight, and preterm birth^55,56,57^ as well as altering fetal brain development and later cognitive function.^58,59^ The cumulative impact on pregnancy and subsequent child health are seen in these outcomes throughout the lifespan and can extend across several generations.^60^

With respect to the Healthy Baby Prenatal Benefit, while its original dollar amount is no longer sufficient to meet the current needs of low-income mothers and infants, it could once again support better outcomes if funding was increased periodically to keep pace with inflation. In April 2024, the Manitoba government announced a budget measure to double the amount of the benefit to better support low-income families,^61^ citing evidence that early and ongoing prenatal care is key to ensuring good outcomes for Manitoba parents and their babies. This is supported by other evidence suggesting that higher funding for social assistance programs correlates with better outcomes. For example, the Canada Child Benefit is an unconditional cash transfer that has been indexed to inflation with beneficial outcomes for families and children, including a decrease in child poverty.^62,63^ A similar indexation for programs like the Healthy Baby Prenatal Benefit could offset inflation and rising costs of living and will, in all likelihood, help to ensure improved population health and well-being. Our future research will include an evaluation of birth and early child development outcomes starting in July 2024, which is when the increase took effect.

### Limitations

The study has some important limitations that warrant mention. Although we leveraged the individual-level sociodemographic information in the Manitoba Population Research Data Repository to adjust for baseline differences between study groups, there is still potential for unmeasured confounders. However, we note that had unmeasured confounders been driving the differences between benefit recipients and nonrecipients, we would not expect to see the associations change over time, lending support to our conclusion that the diminishing purchasing power of the benefit was a prominent contributing factor. We were also limited to data collected in 2019 and earlier. EDI data collection in 2021 was cancelled because of the COVID-19 pandemic, and the 2023 data are not yet available for research. However, given the widespread socioeconomic challenges during the pandemic years, it is doubtful that the trend we observed in this study was reversed during the missing years. Furthermore, we did not have the appropriate partnerships with First Nations and Red River Métis researchers and communities to examine our research question in these populations. From other evaluations of the Healthy Baby Prenatal Benefit we have conducted in partnership with Manitoba First Nations^28^ and Red River Métis,^27^ we know that many of the women included in the study population are Indigenous.

## Conclusions

In this cohort study of low-income mothers and children, the weakening association between the Healthy Baby Prenatal Benefit and positive outcomes suggests that the impact of the benefit has diminished over time. An increase in the benefit amount and periodic adjustments to keep pace with inflation would be necessary to restore and sustain effective support for low-income mothers and infants during a critical period of development. The Healthy Baby Prenatal Benefit has previously proven a valuable strategy for improving birth outcomes, early child development, and population-level health equity. Governments that prioritize providing sufficient resources to families experiencing poverty will see their return on investment in improved population health and well-being. We look forward to evaluating the increased benefit in the coming years.

## Notes

Supplement 1. **eFigure.** Standardized Differences in Study Population Characteristics Before and After Applying Inverse Probability Treatment Weights (in 4-Year Periods From 2003-2019) **eTable 1.** Association of Receiving the Healthy Baby Prenatal Benefit With Birth Outcomes (Risk Ratios) **eTable 2.** Association of Receiving the Healthy Baby Prenatal Benefit With Early Childhood Development (Risk Ratios) **eTable 3.** Association of Receiving the Healthy Baby Prenatal Benefit With Birth Outcomes (Risk Differences) **eTable 4.** Association of Receiving the Healthy Baby Prenatal Benefit With Early Childhood Development (Risk Differences)

Supplement 2.
Data Sharing Statement
