# Supplementary material for: Birth and Early Childhood Outcomes in Families Receiving an Unconditional Prenatal Cash Benefit
Source: JAMA Netw Open. 2025 Aug 14;8(8):e2526996. doi: 10.1001/jamanetworkopen.2025.26996 (PMC12355286; doi:10.1001/jamanetworkopen.2025.26996)
Supplement: Supplement 1. — eFigure. Standardized Differences in Study Population Characteristics Before and After Applying Inverse Probability Treatment Weights (in 4-Year Periods From 2003-2019) eTable 1. Association of Receiving the Healthy Baby Prenatal Benefit With Birth Outcomes (Risk Ratios) eTable 2. Association of Receiving the Healthy Baby Prenatal Benefit With Early Childhood Development (Risk Ratios) eTable 3. Association of Receiving the Healthy Baby Prenatal Benefit With Birth Outcomes (Risk Differences) eTable 4. Association of Receiving the Healthy Baby Prenatal Benefit With Early Childhood Development (Risk Differences) [file jamanetwopen-e2526996-s001.pdf]

Supplemental Online Content

Enns JE, Brownell M, Nickel NC, et al. Birth and early childhood outcomes in families receiving an unconditional prenatal cash benefit. *JAMA Netw Open.* 2025;8(8):e2526996. doi:10.1001/jamanetworkopen.2025.26996

eFigure. Standardized Differences in Study Population Characteristics Before and After Applying Inverse Probability Treatment Weights (in 4-Year Periods From 2003-2019)

eTable 1. Association of Receiving the Healthy Baby Prenatal Benefit With Birth Outcomes (Risk Ratios)

eTable 2. Association of Receiving the Healthy Baby Prenatal Benefit With Early Childhood Development (Risk Ratios)

eTable 3. Association of Receiving the Healthy Baby Prenatal Benefit With Birth Outcomes (Risk Differences)

eTable 4. Association of Receiving the Healthy Baby Prenatal Benefit With Early Childhood Development (Risk Differences)

This supplemental material has been provided by the authors to give readers additional information about their work.

© 2025 Enns JE et al. *JAMA Network Open*.

eFigure. Standardized Differences in Study Population Characteristics Before and After Applying Inverse Probability Treatment Weights (in 4-Year Periods From 2003-2019)

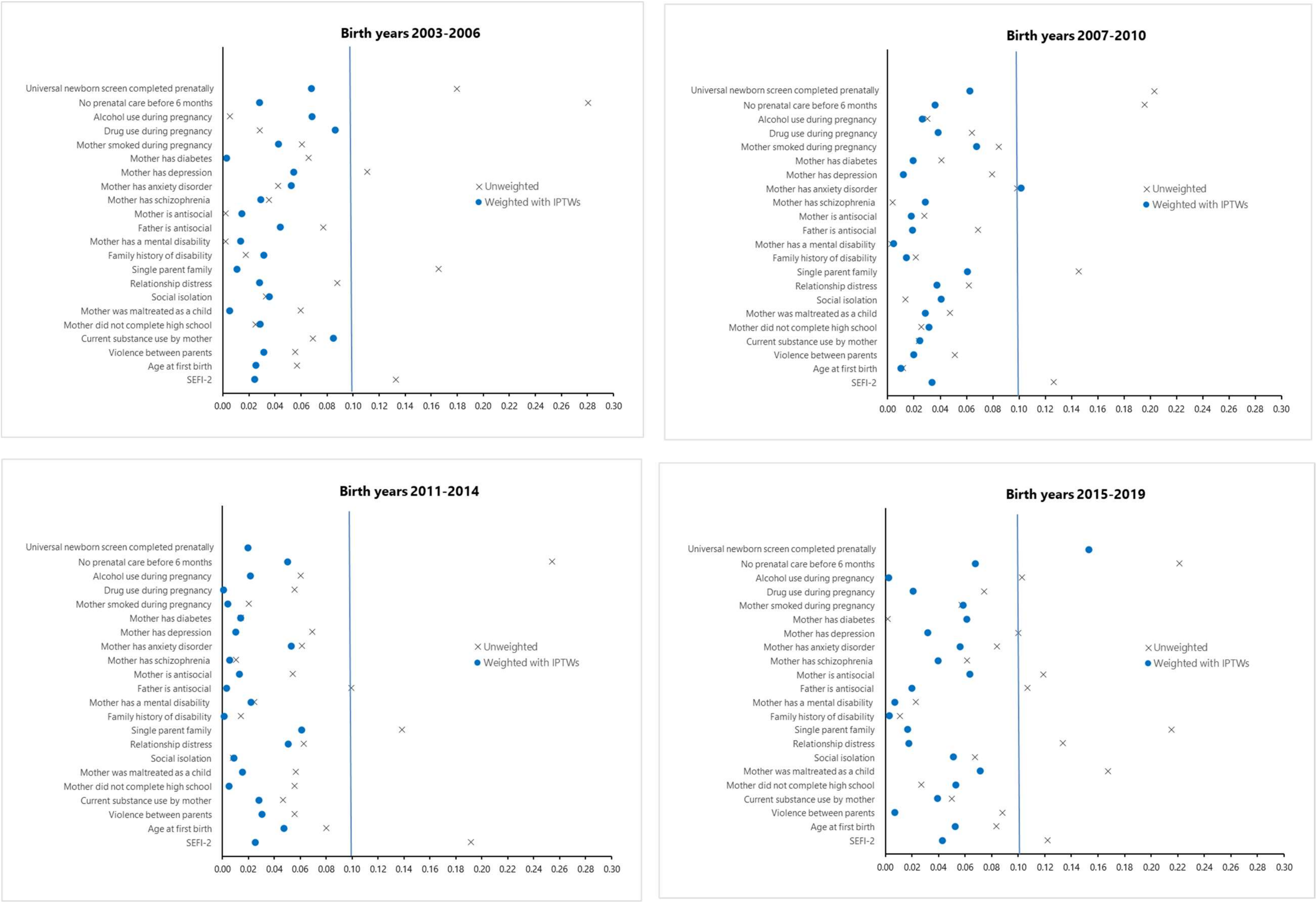

© 2025 Enns JE et al. *JAMA Network Open*.

eTable 1. Association of Receiving the Healthy Baby Prenatal Benefit With Birth Outcomes (Risk Ratios)
Risk ratios comparing the outcomes of low-income mothers who received the benefit to those who did not, 2003-2019

Birth Year	Low Birth Weight RR (95% CI)	Preterm Birth RR (95% CI)	Small for Gestational Age RR (95% CI)	Large for Gestational Age RR (95% CI)	Breastfeeding Initiation RR (95% CI)
2003	0.68 (0.56-0.82)	0.72 (0.62-0.83)	0.83 (0.71-0.96)	1.13 (1.01-1.05)	1.06 (1.02-1.10)
2004	0.69 (0.58-0.82)	0.73 (0.64-0.84)	0.83 (0.73-0.96)	1.13 (1.02-1.05)	1.06 (1.02-1.09)
2005	0.70 (0.60-0.82)	0.74 (0.66-0.84)	0.84 (0.74-0.96)	1.12 (1.03-1.06)	1.06 (1.03-1.09)
2006	0.72 (0.62-0.82)	0.76 (0.68-0.85)	0.85 (0.76-0.95)	1.12 (1.03-1.07)	1.05 (1.03-1.08)
2007	0.73 (0.64-0.83)	0.77 (0.70-0.85)	0.86 (0.78-0.95)	1.12 (1.04-1.07)	1.05 (1.02-1.07)
2008	0.74 (0.66-0.83)	0.78 (0.72-0.86)	0.87 (0.79-0.95)	1.12 (1.05-1.08)	1.05 (1.02-1.07)
2009	0.76 (0.68-0.84)	0.80 (0.73-0.87)	0.88 (0.81-0.96)	1.12 (1.05-1.10)	1.04 (1.02-1.06)
2010	0.77 (0.70-0.85)	0.81 (0.75-0.88)	0.89 (0.82-0.96)	1.12 (1.06-1.12)	1.04 (1.02-1.06)
2011	0.79 (0.71-0.87)	0.83 (0.77-0.89)	0.90 (0.83-0.97)	1.12 (1.06-1.14)	1.04 (1.02-1.06)
2012	0.80 (0.73-0.89)	0.84 (0.78-0.91)	0.91 (0.83-0.98)	1.12 (1.05-1.17)	1.03 (1.02-1.05)
2013	0.82 (0.74-0.91)	0.86 (0.79-0.93)	0.92 (0.84-1.00)	1.12 (1.05-1.21)	1.03 (1.01-1.05)
2014	0.83 (0.74-0.93)	0.87 (0.80-0.95)	0.93 (0.84-1.02)	1.11 (1.04-1.26)	1.03 (1.01-1.05)
2015	0.85 (0.75-0.96)	0.89 (0.81-0.98)	0.93 (0.84-1.04)	1.11 (1.03-1.31)	1.03 (1.00-1.05)
2016	0.87 (0.75-1.00)	0.90 (0.81-1.00)	0.94 (0.84-1.06)	1.11 (1.02-1.37)	1.02 (1.00-1.05)
2017	0.88 (0.76-1.03)	0.92 (0.82-1.03)	0.95 (0.84-1.09)	1.11 (1.01-1.43)	1.02 (0.99-1.05)
2018	0.90 (0.76-1.07)	0.93 (0.82-1.06)	0.96 (0.83-1.12)	1.11 (1.00-1.50)	1.02 (0.99-1.05)
2019	0.92 (0.76-1.11)	0.95 (0.83-1.09)	0.97 (0.83-0.96)	1.11 (0.99-1.58)	1.01 (0.98-1.05)

RR: risk ratio; CI: confidence interval

© 2025 Enns JE et al. *JAMA Network Open*.

eTable 2. Association of Receiving the Healthy Baby Prenatal Benefit With Early Childhood Development
Risk ratios comparing the outcomes of low-income mothers who received the benefit to those who did not, 2003-2019

		Early Development Instrument Domains of Developmental Vulnerability				
**Birth Year**	**Kindergarten Year**	**Physical Health & Well-Being RR (95% CI)**	**Social Competence RR (95% CI)**	**Emotional Maturity RR (95% CI)**	**Language & Cognitive Development RR (95% CI)**	**Communication Skills & General Knowledge RR (95% CI)**
2003	2008/09	0.88 (0.76-1.01)	0.90 (0.76-1.07)	1.00 (0.85-1.18)	0.77 (0.66-0.89)	0.83 (0.72-0.96)
2005	2010/11	0.92 (0.82-1.02)	0.95 (0.83-1.08)	1.00 (0.88-1.13)	0.82 (0.74-0.92)	0.85 (0.76-0.95)
2007	2012/13	0.96 (0.89-1.04)	1.00 (0.90-1.10)	0.99 (0.90-1.09)	0.88 (0.81-0.95)	0.87 (0.80-0.94)
2009	2014/15	1.00 (0.93-1.08)	1.05 (0.96-1.15)	0.99 (0.91-1.08)	0.94 (0.87-1.01)	0.89 (0.82-0.96)
2011	2016/17	1.05 (0.96-1.15)	1.10 (0.99-1.23)	0.99 (0.89-1.10)	1.00 (0.91-1.10)	0.91 (0.83-1.00)
2013	2018/19	1.10 (0.98-1.24)	1.16 (1.00-1.34)	0.99 (0.85-1.14)	1.07 (0.94-1.21)	0.93 (0.82-1.05)

RR: risk ratio; CI: confidence interval

© 2025 Enns JE et al. *JAMA Network Open*.

eTable 3. Association of Receiving the Healthy Baby Prenatal Benefit With Birth Outcomes (Risk Differences)
Risk differences comparing the outcomes of low-income mothers who received the benefit to those who did not, 2003-2019

Birth Year	Low Birth Weight RD (95% CI)	Preterm Birth RD (95% CI)	Small for Gestational Age RD (95% CI)	Large for Gestational Age RD (95% CI)	Breastfeeding Initiation RD (95% CI)
2003	-0.023 (-0.034, -0.011)	-0.029 (-0.043, -0.015)	-0.017 (-0.030, -0.003)	0.018 (0.001, 0.035)	0.039 (0.017, 0.062)
2004	-0.022 (-0.032, -0.011)	-0.028 (-0.040, -0.015)	-0.016 (-0.028, -0.003)	0.018 (0.003, 0.034)	0.038 (0.017, 0.058)
2005	-0.020 (-0.030, -0.011)	-0.026 (-0.037, -0.015)	-0.015 (-0.026, -0.004)	0.018 (0.004, 0.032)	0.036 (0.017, 0.054)
2006	-0.019 (-0.028, -0.011)	-0.025 (-0.035, -0.014)	-0.014 (-0.024, -0.004)	0.018 (0.005, 0.030)	0.034 (0.017, 0.051)
2007	-0.018 (-0.026, -0.011)	-0.023 (-0.032, -0.014)	-0.013 (-0.022, -0.004)	0.018 (0.006, 0.029)	0.032 (0.017, 0.047)
2008	-0.017 (-0.024, -0.010)	-0.022 (-0.030, -0.013)	-0.012 (-0.020, -0.004)	0.018 (0.007, 0.028)	0.030 (0.016, 0.044)
2009	-0.016 (-0.022, -0.010)	-0.020 (-0.028, -0.012)	-0.011 (-0.019, -0.004)	0.018 (0.008, 0.027)	0.028 (0.015, 0.041)
2010	-0.015 (-0.021, -0.009)	-0.019 (-0.026, -0.011)	-0.010 (-0.017, -0.003)	0.017 (0.008, 0.026)	0.026 (0.014, 0.038)
2011	-0.014 (-0.020, -0.008)	-0.017 (-0.024, -0.010)	-0.009 (-0.016, -0.002)	0.017 (0.008, 0.026)	0.024 (0.013, 0.036)
2012	-0.013 (-0.019, -0.007)	-0.015 (-0.023, -0.008)	-0.008 (-0.015, -0.001)	0.017 (0.008, 0.026)	0.022 (0.010, 0.034)
2013	-0.012 (-0.018, -0.005)	-0.014 (-0.022, -0.006)	-0.007 (-0.015, 0.000)	0.017 (0.007, 0.027)	0.020 (0.008, 0.033)
2014	-0.010 (-0.017, -0.004)	-0.012 (-0.021, -0.004)	-0.007 (-0.015, 0.001)	0.017 (0.006, 0.028)	0.019 (0.005, 0.032)
2015	-0.009 (-0.017, -0.002)	-0.011 (-0.021, -0.001)	-0.006 (-0.015, 0.003)	0.017 (0.005, 0.029)	0.017 (0.001, 0.032)
2016	-0.008 (-0.017, 0.000)	-0.009 (-0.020, 0.002)	-0.005 (-0.015, 0.005)	0.017 (0.004, 0.030)	0.015 (-0.002, 0.032)
2017	-0.007 (-0.017, 0.002)	-0.008 (-0.020, 0.004)	-0.004 (-0.015, 0.007)	0.017 (0.002, 0.031)	0.013 (-0.006, 0.032)
2018	-0.006 (-0.017, 0.005)	-0.006 (-0.020, 0.007)	-0.003 (-0.015, 0.009)	0.017 (0.001, 0.032)	0.011 (-0.010, 0.032)
2019	-0.005 (-0.017, 0.007)	-0.005 (-0.020, 0.010)	-0.002 (-0.015, 0.011)	0.016 (-0.001, 0.034)	0.009 (-0.014, 0.032)

RD: risk difference; CI: confidence interval

© 2025 Enns JE et al. *JAMA Network Open*.

eTable 4. Association of Receiving the Healthy Baby Prenatal Benefit With Early Childhood Development (Risk Differences)
Risk differences comparing the outcomes of low-income mothers who received the benefit to those who did not, 2003-2019

		Early Development Instrument Domains of Developmental Vulnerability				
**Birth Year**	**Kindergarten Year**	**Physical Health & Well-Being RD (95% CI)**	**Social Competence RD (95% CI)**	**Emotional Maturity RD (95% CI)**	**Language & Cognitive Development RD (95% CI)**	**Communication Skills & General Knowledge RD (95% CI)**
2003	2008/09	-0.038 (-0.080, 0.003)	-0.023 (-0.061, 0.014)	0.000 (-0.038, 0.038)	-0.070 (-0.111, -0.030)	-0.050 (-0.091, -0.010)
2005	2010/11	-0.024 (-0.055, 0.007)	-0.012 (-0.040, 0.017)	-0.001 (-0.029, 0.028)	-0.053 (-0.083, -0.022)	-0.045 (-0.075, -0.014)
2007	2012/13	-0.010 (-0.034, 0.013)	0.000 (-0.021, 0.022)	-0.001 (-0.023, 0.020)	-0.035 (-0.058, -0.012)	-0.039 (-0.063, -0.016)
2009	2014/15	0.003 (-0.020, 0.027)	0.012 (-0.009, 0.033)	-0.002 (-0.023, 0.019)	-0.017 (-0.040, 0.005	-0.034 (-0.056, -0.011)
2011	2016/17	0.017 (-0.012, 0.047)	0.024 (-0.003, 0.051)	-0.003 (-0.030, 0.024)	0.000 (-0.029, 0.029)	-0.028 (-0.057, 0.001)
2013	2018/19	0.031 (-0.009, 0.071)	0.036 (-0.000, 0.071)	-0.004 (-0.040, 0.032)	0.018 (-0.021, 0.056)	-0.022 (-0.061, 0.016)

RD: risk difference; CI: confidence interval

© 2025 Enns JE et al. *JAMA Network Open*.
