# Supplementary material for: Birth and Early Childhood Outcomes in Families Receiving an Unconditional Prenatal Cash Benefit
Source: JAMA Netw Open. 2025 Aug 14;8(8):e2526996. doi: 10.1001/jamanetworkopen.2025.26996 (PMC12355286; doi:10.1001/jamanetworkopen.2025.26996)
Supplement: Supplement 2. — Data Sharing Statement [file jamanetwopen-e2526996-s002.pdf]

Data Sharing Statement

Enns. Birth and Early Childhood Outcomes in Families Receiving an Unconditional Prenatal Cash Benefit. *JAMA Netw Open*. Published August 14, 2025.
doi:10.1001/jamanetworkopen.2025.26996

Data
Data available: No

Additional Information
Explanation for why data not available: The source data used in this study were originally collected during the routine administration of health and social services in Manitoba, and were provided to the Manitoba Centre for Health Policy (MCHP) for secondary use in research under specific data sharing agreements between the data trustees and MCHP. The data are approved for use at MCHP only. They are not owned by the researchers or by MCHP and cannot be deposited in a public repository. To review source data specific to this article or project, interested parties should contact the MCHP Repository Access & Use team at MCHP.Access@umanitoba.ca. The team will then facilitate data access by seeking the consent of the original data holders and the required privacy and ethics review bodies on behalf of the party requesting access.
